# Identifying Shared Genetic Structure Patterns among Pacific Northwest Forest Taxa: Insights from Use of Visualization Tools and Computer Simulations

**DOI:** 10.1371/journal.pone.0013683

**Published:** 2010-10-29

**Authors:** Mark P. Miller, Susan M. Haig

**Affiliations:** 1 Department of Biology and Center for High Performance Computing, Utah State University, Logan, Utah, United States of America; 2 Forest and Rangeland Ecosystem Science Center, United States Geological Survey, Corvallis, Oregon, United States of America; BC Centre for Excellence in HIV/AIDS, Canada

## Abstract

**Background:**

Identifying causal relationships in phylogeographic and landscape genetic investigations is notoriously difficult, but can be facilitated by use of multispecies comparisons.

**Methodology/Principal Findings:**

We used data visualizations to identify common spatial patterns within single lineages of four taxa inhabiting Pacific Northwest forests (northern spotted owl: *Strix occidentalis caurina*; red tree vole: *Arborimus longicaudus*; southern torrent salamander: *Rhyacotriton variegatus*; and western white pine: *Pinus monticola*). Visualizations suggested that, despite occupying the same geographical region and habitats, species responded differently to prevailing historical processes. *S. o. caurina* and *P. monticola* demonstrated directional patterns of spatial genetic structure where genetic distances and diversity were greater in southern versus northern locales. *A. longicaudus* and *R. variegatus* displayed opposite patterns where genetic distances were greater in northern versus southern regions. Statistical analyses of directional patterns subsequently confirmed observations from visualizations. Based upon regional climatological history, we hypothesized that observed latitudinal patterns may have been produced by range expansions. Subsequent computer simulations confirmed that directional patterns can be produced by expansion events.

**Conclusions/Significance:**

We discuss phylogeographic hypotheses regarding historical processes that may have produced observed patterns. Inferential methods used here may become increasingly powerful as detailed simulations of organisms and historical scenarios become plausible. We further suggest that inter-specific comparisons of historical patterns take place prior to drawing conclusions regarding effects of current anthropogenic change within landscapes.

## Introduction

Landscape genetic and phylogeographic investigations generally rely on a three-step process: data generation, pattern identification, and pattern explanation. Though data generation is generally a straightforward process, the latter two stages can be difficult in many scenarios. Most genetic studies of natural populations produce large, complex data sets (i.e., data are obtained from relatively large numbers of organisms at numerous distinct spatial locations). Challenges subsequently ensue when attempting to identify inherent quantitative signals (i.e., patterns) that provide insights regarding key historical and recent factors that have shaped contemporary genetic structure. Once identified, the set of putative causal mechanisms that may explain said patterns must likewise be discerned. Identification of causal mechanisms becomes notoriously difficult in most cases due to the non-experimental (non-manipulative) nature of investigations and the fact that only a single realization of the evolutionary process can be observed for a given species in a specific geographical region.

Despite the challenges associated with identification of causal factors, researchers can nonetheless choose among a wide array of computer programs and analytical procedures that facilitate the inferential process [1]. For example, there are well-established phylogenetic reconstruction techniques that can assist with identifying geographical distributions of different lineages, as well as analytical tools that can be used to make inferences about historical or currently ongoing demographic processes [2]–[5] or infer timings and parameters of hypothesized historical events [6]–[9]. Furthermore, the well-known Nested Clade Phylogeographic Analysis (NCPA) approach [10] nominally permits inference of diverse historical processes, though the validity of the approach has become a controversial topic [11]–[18]. More recently, Bayesian phylogeographic methods have generated substantial interest [19], [20], though criticisms of such approaches also exist [21].

When analyses identify patterns within data sets, causal relationships can nonetheless still be difficult to identify due to the fact that different processes can plausibly produce similar qualitative and quantitative signals [22]. However, when concordant patterns are observed among genes, populations, or perhaps most importantly, species, researchers are more likely to be able to derive concrete links between observed patterns and the processes that have produced them. A key precursor to this process involves first identifying the most salient shared patterns in the data that most directly reflect underlying causal mechanisms.

**Figure 1:**
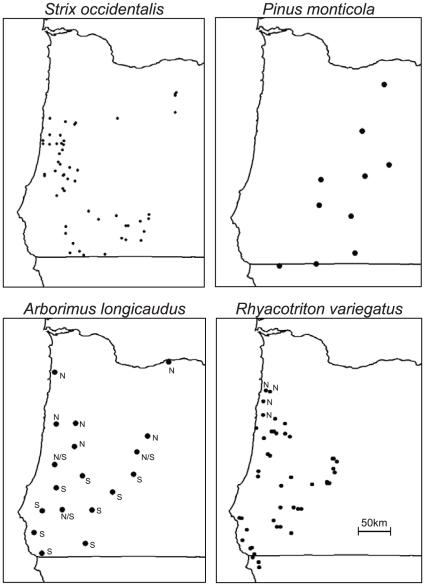
Maps illustrating geographical locations of individuals analyzed for this study. Note that in the case of *S. occidentalis caurina* and *P. monticola*, genetic data reflected the presence of a single phylogenetic lineage or phenetic cluster within the geographical range displayed. In contrast, previous genetic analyses of *A. longicaudus* and *R. variegatus* indicated the presence of separate “northern” and “southern” haplotype lineages in western Oregon (labeled with ‘N’ and ‘S’, respectively). For our analyses, only haplotypes corresponding to “southern” lineages were included in analyses of these two species (unlabeled locations on *R. variegatus* map and locations labeled with an “S” on *A. longicaudus* map). See text for more information.

In this study, we used a four-stage process to help identify and explain shared patterns of spatial genetic structure in four taxa occupying the same habitat in space and time. First, we used a recently-described visualization technique (the “Genetic Landscape Shape” procedure [23], [24]) to explore spatial genetic structure heterogeneity in four species inhabiting similar mature conifer forest habitats in western Oregon and (in some cases) extreme northern California, USA (Fig. 1). Visualizations identified two distinct directional (latitudinal) spatial genetic structure patterns among the four species examined. Second, we confirmed patterns identified in our visualizations via post hoc statistical analysis. Third, based on the observed latitudinal patterns, we developed hypotheses and conceptual models implicating recent post-Pleistocene climate change (and the potential for range expansions) as a plausible causal factor. Finally, we implemented computer simulations to confirm that directional spatial genetic structure patterns can be produced by range expansions. Though in need of additional testing, we also consider alternate phylogeographic hypotheses regarding historical processes that may have produced observations.

## Materials and Methods

### Data set construction

The goal of this investigation was to explore patterns of spatial genetic structure within single phylogenetic lineages of four different species. Four published data sets were used that met three important criteria: 1) they included reasonable levels of sampling within our region of interest, 2) longitude/latitude or zone 10 UTM coordinates of each sampling location were available, and 3) distinct, single phylogenetic-lineage data sets could be defined for each taxon within the region. Studies meeting these criteria included analyses of a bird (*Strix occidentalis caurina*), a tree vole (*Arborimus longicaudus  =  Phenacomys longicaudus*), a salamander (*Rhyacotriton variegatus*), and a tree (*Pinus monticola*).

*S. o. caurina* data were based on DNA sequences from a 522 bp region of the mitochondrial d-loop [25]. We analyzed data from 21 unique haplotypes detected in 58 individuals collected at 55 different locations (Fig. 1). *S. o. caurina* haplotypes comprised a single phylogenetic lineage with an average p-distance of 0.007. No birds previously identified as subspecific hybrids [26] were included in analyses. Data from *P. monticola* were based on 12 isozyme loci scored for an average of 26.9 individuals per population at each of 10 locations (Fig. 1: [27]). Genetic distances [28] among *P. monticola* collection locations (locations 23, 25, 26, 27, 28, 29, 30, 31, 32, and 33 as reported in Table 5 of [27]) were used in our analyses (see below). Other *P. monticola* collection locations examined in that study were either outside the range of our geographical region of interest or part of a highly divergent cluster of four populations from central California or extreme south central Oregon (See Fig. 2 in [27]).

**Figure 2:**
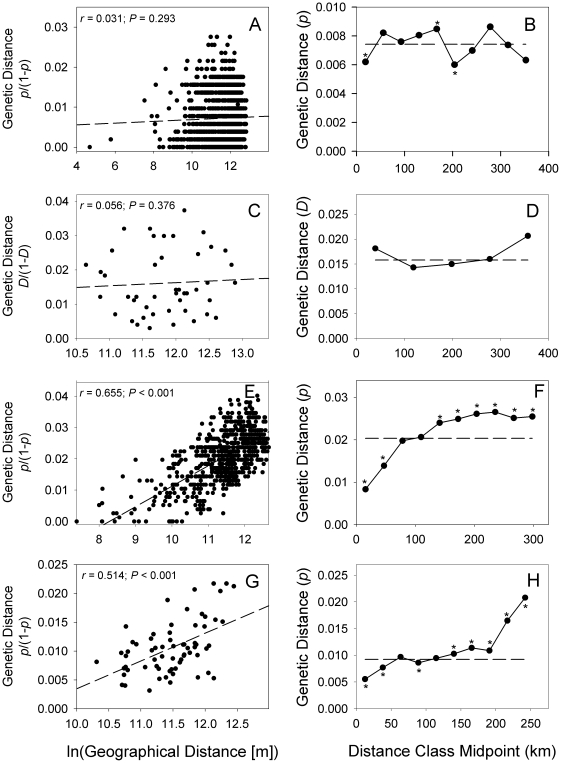
Results of Mantel tests (left column) and spatial autocorrelation analyses (right column) for four species investigated. Panels A and B: *S. o. caurina*. Panels C and D: *P. monticola*, Panels E and F: *R. variegatus*, Panels G and H: *A. longicaudus*. Distance classes in spatial autocorrelations that showed significantly larger or smaller values at the α  = 0.05 level than average (indicated by horizontal dashed lines) are marked with asterisks.

Previous analyses of *R. variegatus* revealed two distinct lineages in our study region that corresponded to separate “northern Oregon” and “central Oregon” clades [29]. Haplotypes from the northern clade were found only at four geographically proximate locations in northern coastal Oregon (Fig. 1). Given our goal of characterizing spatial genetic structure within lineages, “northern” haplotypes from these locations were excluded from analyses, as the small number and close proximity of these sites were not conducive to spatial statistical examination. Consequently, our final data for *R. variegatus* included 34 haplotypes observed in 111 specimens collected at 42 distinct collection locations (Fig. 1). Data were based on a 778 bp region of the mitochondrial cytochrome oxidase subunit I gene, and all included haplotypes were part of a single “central Oregon” phylogenetic lineage with an average p-distance of 0.020.

Analyses of *A. longicaudus* were based on DNA sequences from a 400 bp region of the mitochondrial control region [24]. As with prior analyses of *R. variegatus*, analyses of *A. longicaudus* revealed two distinct evolutionary lineages in western Oregon that were in a few cases found within the same sampling areas (Fig. 1, [24]). For consistency with our data from *R. variegatus*, we restricted our analyses of *A. longicaudus* to those 100 individuals from 12 sampling locations where the 40 “southern” haplotypes were detected (average p-distance  =  0.009). All data for “northern” haplotypes were excluded in our new analyses.

### Data analyses

All data sets were initially analyzed via Mantel tests and spatial autocorrelation as implemented in the computer program Alleles In Space [23]. (Not all analysis variants presented here are available in the current release of Alleles In Space. Analyses were in some cases performed using a new program source code branch in order to address reviewer comments. However, qualitatively and quantitatively similar analysis results were also obtained when using default program options available in the currently-distributed version of AIS.) These procedures provided us with a conventional view of spatial genetic structure within each species by quantifying average structure patterns across our geographical region of interest [30]. Genetic distances used in analyses were calculated as the proportion of mismatched nucleotide sites (*p*) between individuals for *S. o. caurina*, *A. longicaudus*, and *R. variegates*. For *P. monticola*, Genetic distances (*D*) listed in Table 5 of [27] were used. In Mantel tests, 5000 randomization replicates were used to evaluate the significance of correlations between *p*/(1 – *p*) or *D*/(1 – *D*) and the natural logarithm of geographic distance (*sensu* [31]). For spatial autocorrelation analyses, ten distance classes were used with the exception of the *P. monticola* data set. Due to the relatively low number of sampling locations available for *P. monticola* (Fig. 1), five distance classes were used to ensure that all distance classes had observations for statistical analysis. Five thousand randomization replicates were used to identify distance classes with significantly smaller or larger average genetic distance values than expected at random.

Data were subsequently analyzed using the “Genetic Landscape Shape” procedure [24] in AIS. This procedure yields an interpolation-based graphical depiction of spatial genetic structure and diversity across landscapes that can be used to identify genetic discontinuities or landscape regions where relatively high or low genetic distances exist. The procedure was performed by initially generating a Delaunay triangulation-based connectivity network for each species' set of sampling locations (see Fig. 2 in [24]). Next, genetic distances between sampling locations were calculated as described above (proportion of mismatched nucleotides: *p*) in analyses of *S. o. caurina*, *A. longicaudus*, and *R. variegatus*. Nei's genetic distances (*D*) listed in Table 5 of [27] were used for the *P. monticola* data set. Given that there was variation in the geographical distances between sampling locations (Fig. 1), we used residual genetic distances derived from the linear regression of pairwise genetic distances [*p*/(1 – *p*) or *D*/(1 – *D*)] versus the natural logarithm of geographical distances in analyses. Theoretically, this approach accounted for correlations between genetic and geographical distances that may be present and ensured that large interpolation peaks were not resolved solely due to the fact that one or a few sampling areas were geographically isolated [32]. We note that when no correlations between genetic and geographical distances exist, residual genetic distances nonetheless will preserve the relative magnitudes of differences in raw genetic distances present in a data set. Residual genetic distances were assigned to midpoints of connectivity network edges and used throughout the remainder of the Genetic Landscape Shape interpolation procedure (See Fig. 2 in [24]). Final interpolated surfaces were produced using a 50×50 grid and a distance weighting value (a) of 0.1.

Our Genetic Landscape Shape visualizations suggested distinct directional patterns of spatial genetic structure along a North/South axis (see Results). To formally validate these patterns, we performed a series of post-hoc randomization analyses to assess the correlation between genetic distance patterns and latitude [24]. The analysis approach used UTM Northing as one variable and residual genetic distances obtained from the linear regression of genetic distance on geographical distances as the second variable. 5000 randomization replicates were used to evaluate P-values.

### Simulations

The North-South directional patterns observed in our empirical data sets, coupled with established knowledge of historical climate change in the region, led us to hypothesize that range expansion events may have been a primary factor influencing observed spatial genetic structure patterns (see Discussion). To investigate this process, we adopted a two-stage simulation procedure to explore spatial genetic patterns produced during range expansions. In the first stage, a spatially structured population was allowed to evolve until patterns of diversity reached a quasi-equilibrium state reflecting expected number of alleles and heterozygosity based on the simulated population size and specified mutation rate (µ). In the second stage, the simulated population was allowed to extend its range into previously unoccupied landscape regions. The general procedure encapsulated and generalized core processes of evolving spatially structured populations: 1) organisms inhabit a landscape, 2) each organism is born at a landscape location, 3) organisms reproduce (leave progeny) at any number of landscape locations, 4) distances between birth and breeding sites are a function of dispersal ability, 5) progeny genomes are inherited from parents, and 6) alleles inherited from parents can mutate. The approach was similar to that of Slatkin and Barton's lattice model [33] modified to simulate processes at a 500 bp DNA segment evolving under an unconstrained model of sequence evolution. At simulation onsets, all individuals harbored identical homozygous genotypes. Over the course of many generations, the number of alleles and heterozygosity of the systems evolved to reflect theoretical expectations based on specified numbers of simulated organisms and mutation rates (µ) [34]. For our work, we assumed that organismal movement followed a bivariate normal distribution with parameter σ. Consequently, the mean axial distance between individual birth and breeding sites (δ) is δ  =  σ√(π/2) with a standard deviation of υ  =  σ√(2- π/2) [35]. We initially performed simulations of a 100×100 landscape (10,000 simulated organisms). In total, four separate combinations of dispersal distances and mutation rates were explored: δ = 1.5, µ = 10^−4^; δ = 10.0, µ = 10^−4^; δ = 1.5, µ = 2.5*10^−5^; δ = 10.0, µ = 2.5*10^−5^. Note that through manipulation of the above expressions, our dispersal parameters nominally correspond to values of Wright's neighborhood sizes [36] (Ns = 4 πυ^2^) of 18 and 800 for δ  = 1.5 and δ = 10, respectively. Twenty-five simulation replicates of each parameter combination were performed. In the initial phase, simulations were allowed to run for 25,000 generations (µ = 10^−4^) or 85,000 generations (µ = 2.5*10^−5^) to permit quasi-equilibrium patterns of spatial genetic structure and diversity to evolve. In the case of a population of 10,000 individuals and µ = 10^−4^, population genetic theory predicts an expected number of alleles (*a*) and heterozygosity (*H*) to be 34.59 and 0.8, respectively, at equilibrium (See eqns. 3.7 and 3.20 in [34]). Likewise, in the µ = 2.5*10^−5^ case, *a* and *H* are expected to be 10.48 and 0.5, respectively. After the first simulation phase, data were checked to ensure that simulated populations reflected theoretical expectations for a and H.

For the second simulation phase, a simple set of rules was developed to regulate the range expansion process. During the range expansion, the initial 100×100 simulated population was assumed to represent a southern refugium for a population that expanded northward into a larger 100×300 landscape (with the upper two-thirds of this landscape initially uninhabited). We followed our normal simulation procedure as described for phase 1, with the exception that empty landscape cells became inhabited during range expansion only when both randomly chosen parental landscape cells were already occupied. For simplicity, we assumed that a newly-occupied cell was always inhabited in all remaining generations. Consequently, over the course of successive simulation generations, the remainder of the landscape was filled at a rate associated with values of δ specified during simulation runs. At two time points during the range expansion event, data sets comprised of a single haplotype from 200 randomly selected individuals were exported for analysis. Ultimately, we examined time points where 1) the lower two-thirds of the landscape (a 100×200 region) was inhabited, and 2) the landscape was completely inhabited. On average, simulations using δ  = 1.5 required 424.21 generations (S.D. = 6.81) and 824.53 generations (S.D. = 12.53) to reach the two-thirds full and completely inhabited states, respectively. Likewise, simulations using δ  = 10 required 62.63 generations (S.D. = 1.07) and 110.78 generations (S.D. = 1.44) to reach the two-thirds full and completely inhabited states, respectively.

Once data were generated, AIS was used to analyze simulated data sets using the Genetic Landscape Shape procedure. Resulting surface plots revealed directional patterns of spatial genetic structure where inter-individual genetic distances were smaller in the expansion zone relative to the southern refugial region (see Results and Discussion). Consequently, simulated data were also analyzed using the randomization-based procedure used for empirical data sets (see above). Ultimately, we quantified the proportion of simulation replicates at each time point and for each parameter set where significant negative correlations (at the α  = 0.05 level) were detected between landscape coordinates along a South to North axis and inter-individual genetic distances.

## Results

Mantel tests and spatial autocorrelation analyses suggested strong spatial genetic structure in *A. longicaudus* and *R. variegatus* (Fig. 2). Mantel tests indicated significant correlations between genetic and geographic distances (*R. variegatus*: r = 0.63, P<0.001; *A. longicaudus*: r = 0.36, P<0.001). Likewise, spatial autocorrelations illustrated that pairwise genetic distances were significantly smaller than average over shorter distances and were significantly larger than random expectations as geographic distances increased. In contrast, both analyses suggested that no (or minimal) spatial genetic structure was present for *S. o. caurina* and *P. monticola* (Fig. 2). Non-significant Mantel tests were found in both species. In *P. monticola*, spatial autocorrelation likewise identified no distance classes that deviated from random expectations. For *S. o. caurina*, although the first, fifth, and sixth distance classes showed significant deviations from the mean value, the overall distogram illustrated no monotonically increasing values as geographical distances increased.

**Figure 3:**
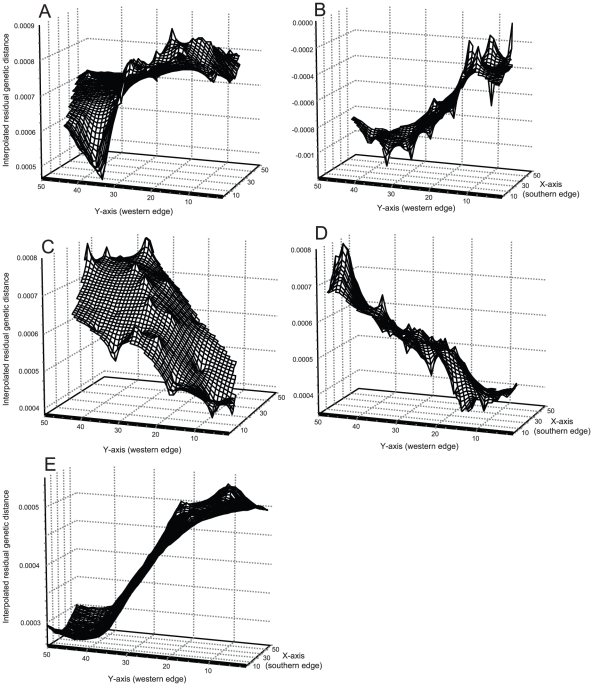
Results of Genetic Landscape Shape interpolations. Panel A: *S. o. caurina*, Panel B: *P. monticola*, Panel C: *A. longicaudus*, Panel D: *R. variegates*, Panel E: a randomly selected simulated data set using δ = 1.5 and µ = 10^−4^ during simulation runs. Surface plot heights reflect genetic distance (and genetic diversity) patterns over the geographical landscape examined.

**Figure 4:**
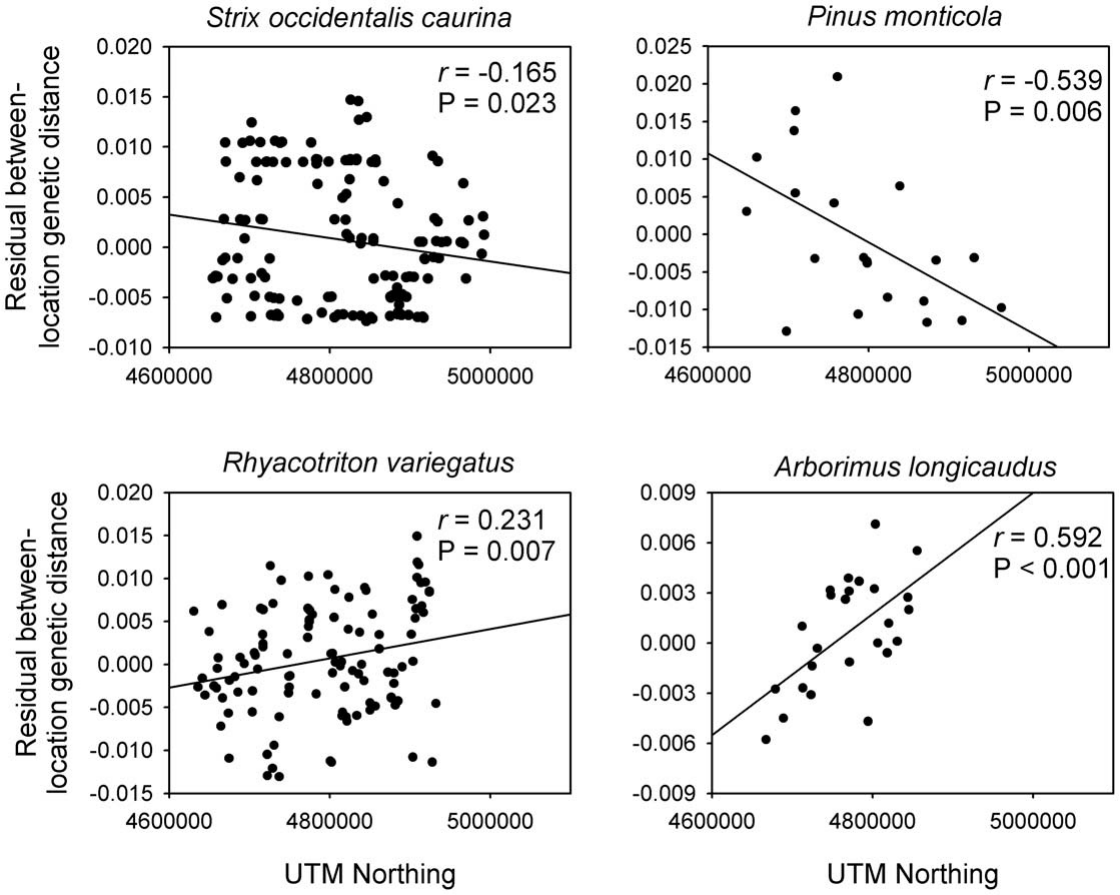
Results of regression/correlation analyses designed to confirm patterns observed in Genetic Landscape Shape analyses (Fig. 3). Analyses of *S. occidentalis caurina* and *P. monticola* show significant negative correlations between genetic distance patterns and UTM northing coordinates. In contrast, *R. variegatus* and *A. longicaudus* both show positive correlations between genetic distance patterns and UTM northing coordinates.

Genetic Landscape Shape visualizations suggested two primary patterns of directional spatial genetic structure and diversity (Fig. 3). In *S. o. caurina* and *P. monticola*, surface plots revealed patterns where larger genetic distances (reflecting higher underlying genetic diversity) were present in the south relative to the north. In contrast, *A. longicaudus* and *R. variegatus* indicated opposite trends. Our *post-hoc* analyses illustrated significant associations between UTM northing and genetic distances in all four species (Fig. 4). Consistent with Fig. 3, correlations for *S. o. caurina* and *P. monticola* were negative (r = −0.167 and r = −0.542, respectively). In contrast, correlations for *A. longicaudus* and *R. variegatus* were positive (r = 0.605 and r = 0.181, respectively).

Simulated range expansions revealed directional patterns whereby inter-individual genetic distances were reduced in the direction of the expansion event (Fig. 3e, Table 1). Analyses of four simulation parameter combinations and two time points revealed several trends (Table 1). First, for a given mutation rate, a higher proportion of data sets produced significant results when dispersal distances were small (δ = 1.5) relative to large (δ = 10). Second, for a given average dispersal distance, more data sets produced significant directional patterns when mutation rates were high (µ = 10^−4^) relative to low (µ = 2.5*10^−5^). Finally, in three of the four parameter combinations, more significant directional patterns were observed after the landscape became completely inhabited relative to the two-thirds inhabited case.

**Table 1:** Proportion of 25 simulated range expansion event replicates where a significant negative correlation at the α  = 0.05 level was observed between landscape coordinates along a South to North transect and pairwise genetic distances.

	Simulation Parameters			
Landscape inhabitation	δ = 1.5, µ = 10^−4^	δ = 1.5, µ = 2.5*10^−5^	δ = 10, µ = 10^−4^	δ = 10, µ = 2.5*10^−5^
Two-thirds full	1.00	0.64	0.60	0.16
Completely full	1.00	0.92	0.64	0.32

The negative correlations indicated that pairwise genetic distances (and diversity) were reduced in the direction of the range expansion event.

## Discussion

Most population genetic and phylogeographic investigations produce large, complex data sets. For example, the (n*(n-1))/2 genetic distances that can be calculated from all pairwise combinations of n samples or sampling locations may generate extremely unwieldy matrices that are difficult to interpret, especially when there is an interest in identifying patterns based on joint analyses of spatial and genetic information. Historically, such data sets have been analyzed using Mantel tests [37] or different forms of spatial autocorrelation analyses [38]. However, these procedures are ideally suited for situations where a homogeneous spatial process is in effect across landscapes, as they characterize average isolation-by-distance patterns within regions [31] and do not identify potentially important heterogeneous patterns of spatial genetic structure that may exist. Numerous analytical procedures have been recently developed to address this issue that provide a basis for identifying cryptic genetic structure, genetic discontinuities/recent vicariance, or maximally-differentiated sets of populations [31], [39]–[43]. While these newer approaches are powerful, they nonetheless only allow researchers to discern between two general alternative scenarios: the presence of a single panmictic “population” or two or more genetically differentiated groups. They do not provide mechanisms for identifying other types of heterogeneous spatial genetic structure that may exist. In this investigation, all four data sets revealed genetic structure patterns of a type that is generally not considered in most investigations. The directional spatial genetic patterns observed here are clearly not appropriate for analysis using models encompassed by the above methods.

### Observed spatial genetic structure patterns

Data sets included in this investigation varied in size, sampling effort, and marker types. Nonetheless, our analyses revealed two main sets of shared patterns among the four species examined. First, results from Mantel tests and spatial autocorrelations differed among species (Fig. 2): *A. longicaudus* and *R. variegatus* showed strong spatial genetic structure, whereas *P. monticola* and *S. o. caurina* revealed no (or little) evidence of such patterns. When visualizations were implemented, an alternate view of spatial genetic structure within these species emerged. Interestingly, the overt patterns observed were of a type not generally considered in most investigations: shared directional patterns of genetic structure existed along a South to North transect (Fig. 3). In *S. o. caurina* and *P. monticola*, genetic distances were reduced in the North relative to the South. Similar patterns have also been observed in microsatellite analyses of *S. o. caurina* from this region (CW Funk and SM Haig, unpublished manuscript). In a regional context, this pattern reflected greater genetic diversity in southern relative to northern locales. In contrast, genetic distances were larger in the North relative to the South for *A. longicaudus* and *R. variegatus*. Although visualizations are qualitative descriptions of spatial genetic structure patterns, additional post-hoc statistical analyses confirmed the graphical depictions generated (Fig. 4). Therefore, just as bivariate scatter plots are commonly used to illustrate relationships between variables prior to choosing appropriate regression models (i.e., linear, polynomial, exponential, etc.), Genetic Landscape Shape interpolations may be a valuable tool for identifying general spatial genetic structure models that can be subsequently quantified and tested via more rigorous statistical methods. Furthermore, such analyses may also be useful for illuminating shared patterns that occur among multiple different species [44]. Remarkably, despite the fact that western Oregon harbors an extremely complex landscape (two mountain ranges, large rivers, low-elevation valleys, and numerous anthropogenic changes to the landscape over the past 50 years), a single linear geographical dimension described the dominant genetic structure pattern in four diverse taxonomic groups (Fig. 4). Based on these findings, an evaluation of potential factors that could lead to the observed latitudinal patterns was in order.

### Genealogical concordance in the Pacific Northwestern U.S

The Pacific Northwest United States has been a common subject of phylogeographic investigations. Over time, a growing body of literature has illustrated interesting congruent patterns among diverse taxonomic groups [29], [45]–[48]. The primary patterns observed among species are shared North/South genetic discontinuities (lower panels of Fig. 1) frequently found in taxa inhabiting western Oregon [47]. Pleistocene glaciation events have been suggested as the most probable mechanistic explanation for many of the observed patterns, and two hypotheses have been proposed to provide specific mechanisms [47]. In the North-South recolonization hypothesis, at least two glacial refugia existed: one south of the glacial maximum and one in non-glaciated northern refugia. After glacial retreat and establishment of more mesic climate regimes, distinct northern and southern lineages expanded their ranges and came into secondary contact to produce observed genetic discontinuities. Under the Leading Edge hypothesis, only individuals South of the glacial maximum persisted during glacial periods. Following glacial retreat, northward range expansions occurred as habitat became suitable for different organisms. Individuals at the most northward “leading edge” of this expansion were primarily responsible for the expansion and carried with them only a subset of the total genetic variation found within the southern refugia. Computer simulations have illustrated that, under some conditions, shallow genetic discontinuities can emerge during range expansions even in the absence of strict geographical barriers [49]. In this investigation, we excluded data from different groups associated with genetic discontinuities (see Methods) and instead focused on data from single lineages. Nonetheless, existing literature led us to hypothesize that observed patterns were caused by range expansions.

### Comparisons of simulated and empirical data under a northern range expansion scenario

Results of our simulation analyses generally illustrated that pairwise genetic distances become reduced in the direction of the range expansion (Fig. 3e, Table 1). This finding is consistent with expectations derived from other simulation investigations [50]. Furthermore, close examination of analysis results revealed three main trends. First, for a given mutation rate, more simulation replicates showed significant directional patterns when dispersal distances were smaller than larger (Table 1). When dispersal was low, only individuals at the northern part of the southern refugium (representing a subset of the total genetic variation) were capable of actively colonizing the expansion zone. Consequently, the range expansion became similar to a series of founder events leading to a situation where individuals in newly-occupied areas were more closely related. In the Genetic Landscape Shape visualization, higher relatedness is reflected by lower inter-individual genetic distances (lower surface plot heights) in the expansion area (Fig. 3e). In the high dispersal cases, there were frequently simulations where no significant directional patterns were observed (Table 1). These replicates indicate that representative genetic diversity from the entire spatially structured southern refugium was present along the expansion leading edge, primarily due to the fact that individuals well-inside the southern refugium were participants in the range expansion process. Second, more replicates showed significant directional patterns at higher mutation rates (relative to a fixed parameterized dispersal distance). In this case, at higher mutation rates, the original southern refugia had higher allelic diversity than their low mutation rate counterparts. When the range expansion occurred, a lower overall proportion of the total genetic variation was carried along the leading edge of the expansion event. Finally, in three out of four parameter sets, more significant directional patterns were observed once the range expansion was completed relative to the time point where only the lower two-thirds of the landscape was inhabited. This result indicates that the ability to detect range expansions may be determined in part by the spatial scale over which the expansion occurs. If the expansion occurs over only relatively small distances, then there will be a reduced chance of detecting directional patterns. In contrast, our data indicate that range expansions are easier to detect when organisms colonize larger geographical regions.

Though our empirical data reflected the same general geographical area, our analyses indicated that different species responded differently to the same historical events and processes that occurred. *S. o. caurina* and *P. monticola* provided the closest qualitative matches to our simulation results (Fig. 3), as both revealed reduced genetic distance patterns in a northward direction (similar to those produced in simulated northern range expansions). In the simplest case, highly mobile species such as birds (*S. o. caurina*) likely avoided cold temperature regimes during glacial periods by actively moving to more suitable southern refugia. As suggested for other avian species [51]–[53], and diverse vertebrates in general [54], *S. o. caurina* likely expanded its range northward following glacial retreat once environmental conditions became favorable. Similarly, we suggest that *P. monticola*, while clearly not capable of avoiding unfavorable climatic conditions, likely was instead extirpated from northern regions of its range during glacial periods. Again, following glacial retreat, the species may have been reestablished in northern areas through wind and animal-mediated seed dispersal. *P. monticola* seeds generally disperse <120 m from parental trees, but have been reported to travel upwards of 800 m in some cases [55]. More importantly, like many other *Pinus* species, mammal and bird species actively feed upon, cache, and disperse *P. monticola* seeds [55] and may represent vectors by which this species engaged in a northern range expansion (especially if said vector species also underwent northern range expansions). Interestingly, though Mantel tests and spatial autocorrelation analyses revealed no significant average spatial genetic structure (Fig. 2), our visualizations (Fig. 3) and quantitative confirmations of observed directional patterns (Fig. 4) indicated that heterogeneous spatial genetic structure existed for these two species. If a range expansion was in fact the primary determinant of the directional trends observed in *S. o. caurina* and *P. monticola*, we suggest that this process may have disrupted a previously-existing isolation-by-distance process and lead to the inability of these two procedures to detect underlying patterns.

*R. variegatus* and *A. longicaudus* displayed patterns opposite to those identified in *S. o. caurina*, *P. monticola*, and the strict northward range expansion scenario encapsulated in simulations (Fig. 3). In these two species, genetic distances were lower in southern regions relative to northern ones. We suggest two plausible explanations for this outcome. First, our analyses may reflect southern range expansions from northern refugia. Indeed, in both species, there exist highly divergent separate clades to the north of the area investigated (Fig. 1; [24], [29]). These observations suggest that the species were nominally capable of persisting within the region investigated, perhaps in warmer coastal refugia, during periods of inhospitable climatic conditions. The plausibility of southern range expansions, however, is predicated upon identification of factors that would have extirpated these species from southern regions while allowing them to persist in northern ones prior to a southern expansion event. Alternately, these findings may reflect different types of historical directional environmental factors. For example, current data indicate that the Cordilleran ice sheet from the Wisconsin glacier (glacial maximum ∼14,000 years ago) did not spread southward far enough to cover the geographical region investigated in this study [56]–[57]. However, climate change associated with the Wisconsin glaciation had dramatic effects on forest communities within western Oregon, which became fragmented by tundra and cold steppes during that time [57]. Given the latitudinal thermal gradient that likely existed (colder temperatures in northern areas closer to the glacial sheet), a gradient of habitat fragmentation may have also been in place that produced greater habitat connectivity in southern regions. If *R. variegatus* and *A. longicaudus* persisted in western Oregon during this time, then increased habitat fragmentation in northern regions may have constrained dispersal among suitable habitat patches and resulted in greater genetic differentiation of northern versus southern populations. Note that both *R. variegatus* and *A. longicaudus* showed significant average patterns of spatial genetic structure (Fig. 2) in addition to the directional patterns observed in our visualizations (Fig. 3). This finding may indicate that these species did not undergo a southern range expansion, and instead may suggest the presence of more long-term and stable spatial evolutionary processes. If latitudinal variation in habitat fragmentation existed during the most recent glaciation, it apparently was not of sufficient magnitude to disrupt the overall isolation-by-distance pattern that currently exists.

### Conclusions

Three of the four species investigated are of conservation importance in the Pacific Northwest United States. The northern spotted owl (*S. o. caurina*) is protected under the U.S. Endangered Species Act as a threatened subspecies [58]. *A. longicaudus* is currently the only mammal protected by the “Survey and Manage” portion of the Northwest Forest Plan due to its importance as a food source for *S. o. occidentalis* [59]. Finally, *R. variegatus* was previously petitioned, but deemed not warranted, for listing under the U.S. Endangered Species Act [60]. Given the conservation importance of these species, there is current interest in determining if various recent human practices (land development, timber harvesting, road construction, etc.) have influenced genetic structure patterns. Recently, analytical procedures have emerged to help infer the effects of various landscape variables on spatial genetic structure [61]. However, our analyses suggested that dominant patterns could be explained by historical processes that are not explicitly tied to landscape features (i.e., non-landscape factors accounted for a large proportion of the variation in spatial genetic structure). In the most dramatic cases, the South to North spatial axis alone explains ∼29% and ∼35% of the variation in genetic distances for *P. monticola* and *A. longicaudus*, respectively (estimated from r^2^ values obtained from squaring correlation coefficients; Fig. 4). This suggests that non-landscape factors that influence spatial genetic structure must be identified prior to invoking anthropogenic or landscape-associated effects [62]. Ultimately, the field of Landscape Genetics may be greatly enhanced by development of new conceptual or analytical frameworks that remove the effects of these factors in analyses, leading to an ability to more clearly identify recent processes that influence overall patterns of spatial genetic structure. Nonetheless, future work designed to infer effects of anthropogenic activities may be enhanced once common shared historical patterns are taken into account. For example, when identifying common multi-species effects of recent disturbances or anthropogenic changes, it may be desirable to perform said analyses on groups of taxa that are categorized based on shared genetic structure patterns as an added control measure. The Genetic Landscape Shape procedure appears to provide a simple and convenient means for achieving this goal.

Debates over the most appropriate tools for genetic studies of natural populations are not likely to end in the near future, particularly when undertaking the challenge of comparing multiple species. We therefore agree with points raised by others regarding use of different integrative analytical tools in a heuristic manner when making inferences [11]. Phylogeography and landscape genetics will increasingly rely on multiple inferential approaches that can be used to refine and test hypotheses about factors that produce observed patterns. In this investigation, data visualizations and spatial simulations were important tools that allowed us to detect and explain patterns observed among diverse taxa. Visualizations played an important role as part of the first step of data exploration, as they facilitated identification of shared patterns of a type not generally considered in most studies. Likewise, scenario-specific simulations allowed us to infer genetic structure patterns produced during range expansion events. Our ability to explore more detailed species-specific historical scenarios should be enhanced as simulation techniques, computational resources, and our understanding of organismal habitat requirements improve in the future [63].

## References

[1] Excoffier L, Heckel G (2006). computer programs for population genetics data analysis: a survival guide.. Nat Rev Genet.

[2] Fu YX (1997). Statistical tests of neutrality against population growth, hitchhiking and background selection.. Genetics.

[3] Fu YX, Li WH (1993). Statistical tests of neutrality of mutations.. Genetics.

[4] Tajima F (1989a). The effect of change in population size on DNA polymorphism.. Genetics.

[5] Tajima F (1989b). Statistical method for testing the neutral mutation hypothesis by DNA polymorphism.. Genetics.

[6] Drummond AJ, Rambaut A, Shapiro B, Pybus OG (2005). Bayesian coalescent inference of past population dynamics from molecular sequences.. Mol Biol Evol.

[7] Excoffier L (2004). Patterns of DNA sequence diversity and genetic structure after a range expansion: lessons from the infinite-island model.. Mol Ecol.

[8] Ray N, Currat M, Excoffier L (2003). Intra-deme molecular diversity in spatially expanding populations.. Mol Biol Evol.

[9] Schneider S, Excoffier L (1999). Estimation of demographic parameters from the distribution of pairwise differences when the mutation rates vary among sites: Application to human mitochondrial DNA.. Genetics.

[10] Templeton AR (1998). Nested clade analyses of phylogeographic data: testing hypotheses about gene flow and population history.. Mol Ecol.

[11] Garrick RC, Dyer RJ, Beheregaray LB, Sunnucks P (2008). Babies and Bathwater: a comment on the premature obituary for nested clade phylogeographic analysis.. Mol Ecol.

[12] Knowles LL, Maddison WP (2002). Statistical phylogeography.. Mol Ecol.

[13] Panchal M, Beaumont MA (2007). The automation and evaluation of nested clade phylogeographic analysis.. Evolution.

[14] Petit RJ (2008a). The coup de grace for the nested clade phylogeographic analysis?. Mol Ecol.

[15] Petit RJ (2008b). On the falsifiablilty of the nested clade phylogeographic analysis method.. Mol Ecol.

[16] Petit RJ, Grivet D (2002). Optimal randomization strategies when testing the existence of a phylogeographic structure.. Genetics.

[17] Templeton AR (2002). “Optimal” randomization strategies when testing the existence of a phylogeographic structure: A reply to Petit and Grivet.. Genetics.

[18] Templeton AR (2008). Nested clade analysis: an extensively validated method for strong phylogeographic inference.. Mol Ecol.

[19] Fagundes NJR, Ray N, Beaumont M, Neuenschwander S, Salzano FM (2007). Statistical evaluation of alternate models of human evolution.. Proc Natl Acad Sci USA.

[20] Lemey P, Rambaut A, Drummond AJ, Suchard MA (2009). Bayesian Phylogeography Finds Its Roots.. PLoS Comput Biol.

[21] Templeton AR (2009). Statistical hypothesis testing in intraspecific phylogeography: NCPA versus ABC.. Mol Ecol.

[22] Nielsen R, Beaumont MA (2009). Statistical inferences in phylogeography.. Mol Ecol.

[23] Miller MP (2005). Alleles In Space: Computer software for the joint analysis of interindividual spatial and genetic information.. J Hered.

[24] Miller MP, Bellinger MR, Forsman ED, Haig SM (2006a). Effects of historical climate change, habitat connectivity, and vicariance on genetic structure and diversity across the range of the red tree vole (*Phenacomys longicaudus*) in the Pacific Northwestern United States.. Mol Ecol.

[25] Haig SM, Mullins TD, Forsman ED (2004). Subspecific relationships and genetic structure in the spotted owl.. Conserv Genet.

[26] Funk WC, Forsman ED, Mullins TD, Haig SM (2008). Introgression and dispersal among spotted owl (*Strix occidentalis*) subspecies.. Evol Appl.

[27] Steinhoff RJ, Joyce DG, Fins S (1983). Isozyme variation in *Pinus monticola*.. Can J Forest Res.

[28] Nei M (1978). Estimation of average heterozygosity and genetic distance from a small number of individuals.. Genetics.

[29] Miller MP, Haig SM, Wagner RS (2006b). Phylogeography and spatial genetic structure of the Southern torrent salamander: Implications for conservation and management.. J Hered.

[30] Manel S, Schwartz MK, Luikart G, Taberlet P (2003). Landscape genetics: combining landscape ecology and population genetics.. Trends Ecol Evol.

[31] Rousset F (1997). Genetic differentiation and estimation of gene flow from F-statistics under isolation by distance.. Genetics.

[32] Manni F, Guérard E, Heyer E (2004). Geographic patterns of (genetic, morphologic, linguistic) variation: how barriers can be detected by “Monmonier's algorithm”.. Hum Biol.

[33] Slatkin M, Barton NH (1989). A comparison of three indirect methods for estimating average levels of gene flow.. Evolution.

[34] Hartl DL, Clark AG (1989). Principles of Population Genetics..

[35] Austerlitz F, Smouse PE (2001). Two-generation analysis of pollen flow across a landscape. II. Relation between Φft, pollen dispersal and interfemale distance.. Genetics.

[36] Wright, S (1946). Isolation by distance under diverse systems of mating.. Genetics.

[37] Mantel N (1967). The detection of disease clustering and a generalized regression approach.. Cancer Res.

[38] Sokal RR, Oden NL (1978). Spatial autocorrelation analysis in biology. 2. Some biological implications and four applications of evolutionary and ecological interest.. Biol J Linn Soc.

[39] Chen C, Durand E, Forbes F, Francois O (2007). Bayesian clustering algorithms ascertaining spatial population structure: a new computer program and a comparison study.. Mol Ecol Notes.

[40] Crida A, Manel S (2007). WOMBSOFT: an R package that implements the Wombling method to identify genetic boundary.. Mol Ecol Notes.

[41] Dupanloup I, Schneider S, Excoffier L (2002). A simulated annealing approach to define the genetic structure of populations.. Mol Ecol.

[42] Guillot G, Estoup A, Mortier F, Cosson JF (2005). A spatial statistical model for landscape genetics.. Genetics.

[43] Manel S, Berthoud F, Bellemain E, Gaudeul M, Luikart G (2007). A new individual-based spatial approach for identifying genetic discontinuities in natural populations.. Mol Ecol.

[44] Vandergast AG, Hathaway SA, Fisher RN, Boys J, Bohonak AJ (2008). Are hotspots of evolutionary potential adequately protected in southern California?. Biol Conserv.

[45] Brunsfeld SJ, Sullivan J, Soltis DE, Soltis PS, Silvertown J, Antonovics J (2001). Comparative phylogeography of northwestern North America: a synthesis.. Integrating ecology and evolution in a spatial context.

[46] Nielson M, Lohman K, Sullivan J (2001). Phylogeography of the tailed frog (*Ascaphus truei*): Implications for the biogeography of the Pacific Northwest.. Evolution.

[47] Soltis DE, Gitzendanner MA, Strenge DD, Soltis PE (1997). Chloroplast DNA intraspecific phylogeography of plants from the Pacific Northwest of North America.. Plant Syst Evol.

[48] Wilke T, Duncan N (2004). Phylogeographical patterns in the American Pacific Northwest: lessons from the arionid slug *Prophysaon coeruleum*.. Molecular Ecology.

[49] Irwin DE (2002). Phylogeographic breaks without geographic barriers to gene flow.. Evolution.

[50] Excoffier L, Ray N (2008). Surfing during population expansion promotes genetic revolutions and structuration.. Trends Ecol Evol.

[51] Burns KJ, Barhoum DN (2006). Population-level history of the wrentit (*Chamaea fasciata*): Implications for comparative phylogeogrphy in the California Floristic Province.. Mol Phylogenet Evol.

[52] Johnson JA (2008). Recent range expansion and divergence among North American prairie grouse.. J Hered.

[53] Míla B, McCormac JE, Castaneda G, Wayne RK, Smith TB (2007). Recent postglacial range expansion drives the rapid diversification of a songbird lineage in the genus *Junco*.. P Roy Soc B-Biol Sci.

[54] Martin PR, McKay JK (2004). Latitudinal variation in genetic divergence of populations and the potential for future speciation.. Evolution.

[55] Graham RT, Burns RM, Honkala BH (2004). Western White Pine.. Silvics of North America.

[56] Andersen BG, Borns HWJ (1994). The ice age world: an introduction to Quaternary history and research with emphasis on North America and Northern Europe during the last 2.5 million years..

[57] Bonnicksen TM (2000). America's ancient forests: from the ice age to the age of discovery..

[58] US Fish and Wildlife Service. (1990). Determination of threatened status for the northern spotted owl.. Federal Register.

[59] USDA Forest Service and USDI Bureau of Land Management (2000). Final supplemental environmental impact statement for amendment to the survey and manage, protection buffer, and other mitigation measures, standards and guides. Vol. 1..

[60] U.S. Fish and Wildlife Service (2000). 12-month finding for a petition to list the southern torrent salamander in California as endangered or threatened.. Federal Register.

[61] Cushman SA, McKelvey KS, Hayden J, Schwartz MK (2006). Gene flow in complex landscapes: Testing multiple hypotheses with causal modeling.. Am Nat.

[62] Balkenhol N, Gugerli F, Cushman SA, Waits LP, Coulon A (2007). Identifying future research needs in landscape genetics: where to from here?. Landscape Ecol.

[63] Epperson BK, McRae BH, Scribner K, Cushman SA, Rosenburg MS (2010). Utility of computer simulations in landscape genetics.. Molecular Ecology.

